# Bibliometric research on the developments of artificial intelligence in radiomics toward nervous system diseases

**DOI:** 10.3389/fneur.2023.1171167

**Published:** 2023-06-09

**Authors:** Jiangli Cui, Xingyu Miao, Xiaoyu Yanghao, Xuqiu Qin

**Affiliations:** Shaanxi Provincial People's Hospital, Xi'an, China

**Keywords:** radiomics, bibliometrics, nervous system diseases, glioblastoma (GBM), deep learning, multi-omics study

## Abstract

**Background:**

The growing interest suggests that the widespread application of radiomics has facilitated the development of neurological disease diagnosis, prognosis, and classification. The application of artificial intelligence methods in radiomics has increasingly achieved outstanding prediction results in recent years. However, there are few studies that have systematically analyzed this field through bibliometrics. Our destination is to study the visual relationships of publications to identify the trends and hotspots in radiomics research and encourage more researchers to participate in radiomics studies.

**Methods:**

Publications in radiomics in the field of neurological disease research can be retrieved from the Web of Science Core Collection. Analysis of relevant countries, institutions, journals, authors, keywords, and references is conducted using Microsoft Excel 2019, VOSviewer, and CiteSpace V. We analyze the research status and hot trends through burst detection.

**Results:**

On October 23, 2022, 746 records of studies on the application of radiomics in the diagnosis of neurological disorders were retrieved and published from 2011 to 2023. Approximately half of them were written by scholars in the United States, and most were published in Frontiers in Oncology, European Radiology, Cancer, and SCIENTIFIC REPORTS. Although China ranks first in the number of publications, the United States is the driving force in the field and enjoys a good academic reputation. NORBERT GALLDIKS and JIE TIAN published the most relevant articles, while GILLIES RJ was cited the most. RADIOLOGY is a representative and influential journal in the field. “Glioma” is a current attractive research hotspot. Keywords such as “machine learning,” “brain metastasis,” and “gene mutations” have recently appeared at the research frontier.

**Conclusion:**

Most of the studies focus on clinical trial outcomes, such as the diagnosis, prediction, and prognosis of neurological disorders. The radiomics biomarkers and multi-omics studies of neurological disorders may soon become a hot topic and should be closely monitored, particularly the relationship between tumor-related non-invasive imaging biomarkers and the intrinsic micro-environment of tumors.

## 1. Introduction

Radiomics was first proposed by PhilippeLambin, a Dutch scholar, in 2012 to measure the shape of tumors and analyze the differences in image texture (1). With the rapid development of medical imaging and artificial intelligence, there has been an increasing number of research in the field of medical image analysis, such as disease prevention, diagnosis, treatment efficacy evaluation, and prognosis prediction (2–7).

In recent years, digital medical imaging has gradually transformed into high-dimensional data suitable for data mining and data science techniques. Aided by powerful computing power and Quantitative Image Analysis (QIA) technologies, radiomics has made rapid development (8). Currently, radiomics and deep learning are the most researched technologies in the field of medical imaging. Radiomics involves high-throughput extraction of a large amount of quantifiable information from regions of interest (ROI) in digital medical images. Deep learning, as a classic artificial intelligence methods, transforms the extractions into hundreds or thousands of quantitative imaging features (9). Then, quantitative extraction and analysis of image features, which roughly include first-order histograms, shape, texture, and wavelets, is performed to establish prediction models for clinical decision support. Unlike traditional computer-aided diagnosis (CAD), radiology focuses on providing information about human diseases from a deep and latent perspective, and the recognition of high-dimensional heterogeneous information in images by radiology is incomparable to traditional CAD.

As the development of Artificial Intelligence (AI) techniques, such as deep learning and convolutional neural networks, it has greatly advanced the performance of computer vision systems (10). These AI methods have enabled vision systems to achieve remarkable results in a wide range of applications, including object detection, video classification, and image segmentation. With the advancement of deep learning algorithms, AI-based methods such as convolutional neural networks (CNNs) (11) and recurrent neural networks (RNNs) (12) have been developed to analyze imaging data and make predictions with high accuracy. Among these applications, AI-based approaches have made preliminary exploration in the field of radiomics. These AI-based radiomics methods have the potential to improve disease diagnosis (13) and treatment decision-making (14), as well as facilitate the development of precision medicine (15).

The workflow of radiomics inspired by AI includes image acquisition and segmentation, radiomics feature extraction, and model building. Once the data is collected and organized, ROI is usually segmented for analysis by qualified professionals such as doctors, either manually or semi-automatically. Some research has shown that the results of automatic image segmentation using deep learning methods are more satisfactory (16, 17). The feature extraction step is noteworthy as different implementations produce different radiomics values, and radiomics models are only applicable to the same feature interpretation. The appearance of the Image Biomarker Standardization Initiative (IBSI) is expected to reduce the impact of this problem (18). Once the image acquisition and radiomics feature extraction are complete, machine learning algorithms can be used to build radiomics models. For example, the software provided by Chen et al. (19) includes the implementation of various modeling algorithms such as decision trees, logistic regression, complex random forests, Bayesian networks, and support vector machines (20–23). These models can also be optimized for specific performance metrics, and the receiver operating characteristic curve (AUC) (6) is widely used for optimization. Another tool to evaluate the models is the calibration plot (24), which describes the relationship between the true sample class and the model prediction probability. Overall, the selection of appropriate modeling algorithms is still an active area of research.

An important reason for the widespread development of radiomics AI methods is that, as the increases of the incidence of neurological diseases and the ratio of disability and death, traditional imaging techniques can only qualitatively describe the lesions, and provide size, shape, and other characteristics which can be similarly recognized by the naked eye. For non-visualized quantitative data such as textures and histograms, direct visualization is not available (25). Radiomics can overcome these shortcomings. Zhang et al. (26) developed an algorithm for fully automatic segmentation of glioblastoma regions in MRI and compared it with a reference established by manual tumor segmentation. The results showed that the algorithm was able to extract most image features with moderate or high accuracy. Meanwhile, Upadhaya et al. (27) attempted to use radiomics for grading and prognostic estimation of glioblastoma multiforme (GBM) and achieved 90% accuracy. Gillies et al. (8) proposed that quantitative features in radiomics include not only imaging features but also clinical and genetic information. This article is currently the most representative review of radiomics research.

In the 2016 World Health Organization Classification of tumors of the central nervous system (WHOC), the key features of the phenotype are combined with genotype, providing new ideas for precise classification, grading, and participation in treatment decision-making of brain tumors (28). The application of machine learning combined with radiomics in the classification, prediction, and prognostic assessment of neurological diseases has increased explosively (29–31). Researchers have gradually shifted their focus to improving the reproducibility of radiomics features, standardizing MRI, and multi-index joint diagnosis, prediction, and prognosis (32–34).

With the continuous progress of radiomics in the study of central nervous system diseases, it is crucial to understand the new trends and key milestones in related knowledge development. However, few systematic analysis have been carried out on these publications. Bibliometrics analysis has been widely used to organize knowledge structures and explore the trends of extensive research fields, including quantitative analysis of patterns in the scientific literature (19). Several studies have shown that CiteSpace focuses on finding the key points in the development of a domain, especially key turning points. Due to its rich functionality, it has become an effective method for analyzing big data at present (35, 36). To the best of our knowledge, there has been no systematic bibliometric analysis of radiomics in neurological disease research. Therefore, we will describe the scientific results of radiomics in the study of central nervous system diseases to identify trends and hotspots, and guide the future work of researchers.

## 2. Methods

### 2.1. Search strategy

The data was downloaded from the Science Citation Index Extended database of the Web of Science Core (WoSCC) on October 26, 2022. The following search terms were used: (“radiomics”) and (“brain” or “cerebrum” or “encephalon” or “pericranium” or “cerebral*” or “central nervous system”) in the Topic field, including title, abstract, author keywords, and KeyWords Plus following continuing of existing search methods (37–39). Original articles and reviews written and published in English between 2011 and 2022 were included. The result of the survey was 764 records, which were obtained from this study.

### 2.2. Data collection and analysis

All records retrieved from WoSCC were downloaded independently by two authors, including the number of publications published annually; country/region, institution, journal, and author output; citation frequency; and H-index. The H-index represents the number of academic journals or scholars/countries/regions that have published H papers, each of which has been cited at least H times It is used to evaluate the scientific impact of authors or countries. Journal Citation Reports (JCR) 2022 was used to obtain the impact factor (IF) of journal categories. The data were then transformed into Microsoft Excel 2019, VOSviewer, and CiteSpace V for the analysis of basic indicators. Microsoft Excel (v.2019) was used to analyze and organize the data on the basic characteristics of publications and citations by plotting the annual publication output, H-index, total IF, and average IF, citation count per article, and total citation count for each country/region.

VOSviewer (36) was used to create network visualization maps to analyze the collaboration among countries/regions, institutions, and highly cited reference authors. Furthermore, VOSviewer can categorize keywords with high co-occurrence frequency into multiple clusters, and color them simultaneously according to the timeline. Co-occurrence analysis determines the research hotspots and trends. We choose “author keywords” as the unit of analysis.

We use CiteSpace V for a merged analysis of journals, references, and clusters, and further constructed a merged reference timeline view, through which the rise and development periods of some cluster fields can be better understood. In addition, CiteSpace can capture keywords with strong reference outbreaks, and construct a visualization map for all projects. Citation burst is a key indicator for recognizing emerging trends (19). We set the “number of years per slice” and “number of years before each slice” to 1 and 50, respectively. Thus, the network map is extracted from the top 50 references cited in the first year of each article.

## 3. Results

### 3.1. Publication output and temporal trend

**Figure 1:**
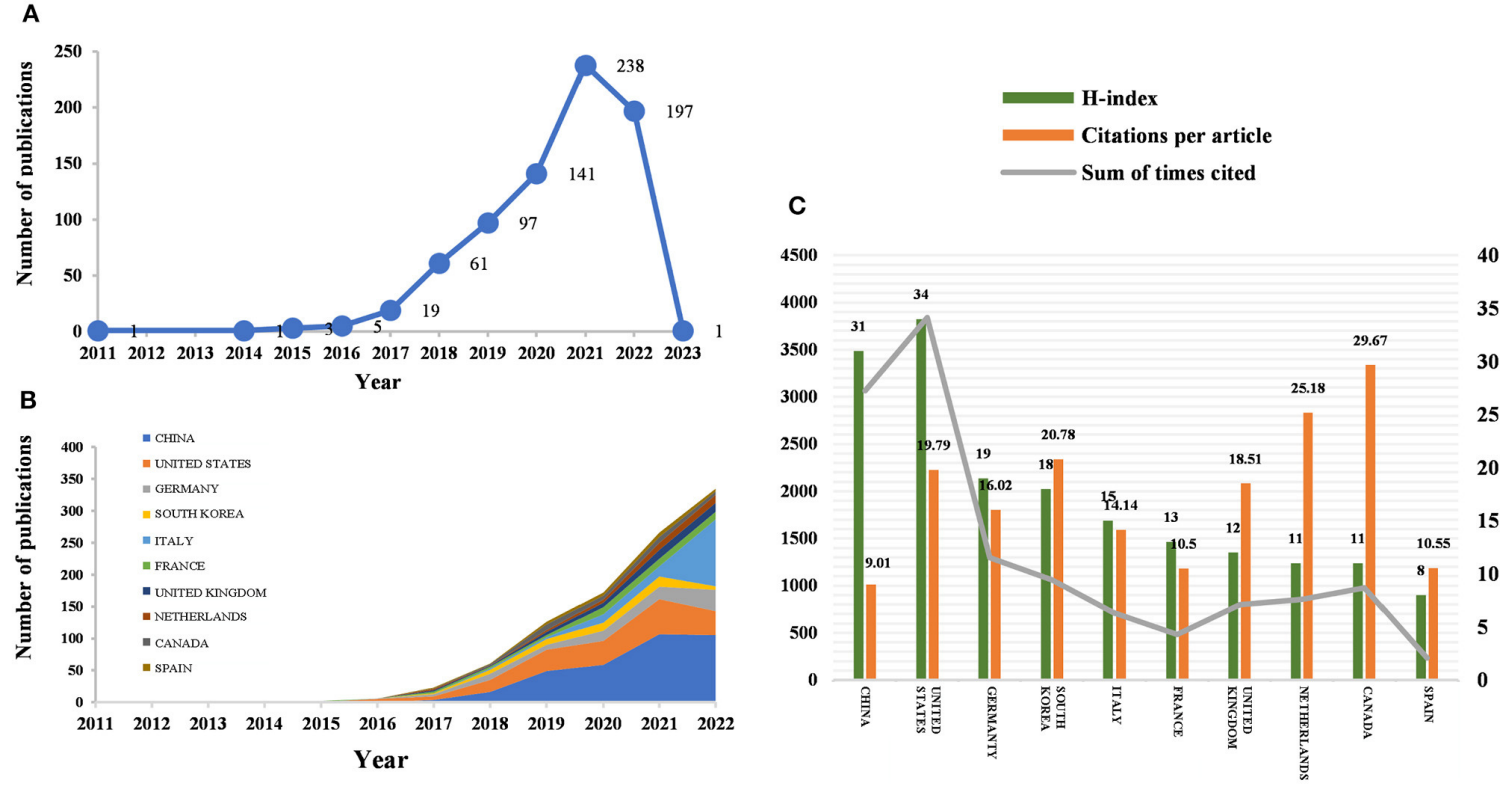
Number of publications and analysis trends of radiomics countries/regions in neurological disease research. **(A)** Annual global publication output. **(B)** Trends in publication output growth for the top 10 countries. **(C)** H-index, number of citations per article, and total number of citations in the top 10 countries/regions.

According to the citation analysis from the Web of Science, a total of 764 publications met the inclusion criteria, consisting of 647 articles and 117 reviews (Figure 1A). In the early stage of research, the average annual citation frequency of published articles was low due to the lack of research on the application of radiomics in neurological diseases. From 2011 to 2017, the field was in a low-heat period with fewer than 30 articles of related research reports. However, from 2018 to 2022, the number of articles published on radiomics in the field of neurological diseases has been steadily increasing, with 61 articles published in 2018 alone, surpassing the previous total. The situation indicates the increasing attention in this field. Since 2018, the average annual citation frequency of published articles has rapidly increased and stabilized at around 300 per year, further indicating that the study of radiomics in neurological diseases has reached a more mature stage. The reason may be due to an increase in the number of neurological disease patients worldwide, as well as the result of interdisciplinary fusion and continuous innovation. In the past 3 years, the number of papers published in this field has shown a clear upward trend, with more than 90 papers published each year appearing in highly active states. With an average annual increase of 70 papers and an average annual growth rate of 57.43%, this indicates that research in this field has received increasing attention. While 197 articles have been published in 2022 so far, this number does not reflect the total number of publications throughout the year. To date, these articles have been cited 10,187 times, averaging 13.3 citations per article.

### 3.2. Distribution by country/region and institution

Between 2011 and 2023, all publications were published in 49 countries/regions and 1,291 institutions (Table 1). China has the most publications (194, 44.503%), followed by the United States (81, 25.393%), South Korea (51, 6.675%), Italy (50, 6.545%), and France (46, 6.021%). With the exception of the United States, all other countries have been published after 2017. Since 2018, China has ranked first in annual publication volume. However, among the five most productive countries, China has the lowest average IF. The annual growth rate of publications produced by the United States and Germany also follows a similar trend. Since 2020, although Italy's cumulative publication count from 2011 to 2022 ranked fifth (50), its annual publication output has grown rapidly exceeding China's by 2022. The total trend of the published numbers by these countries each year shows that since 2017, the number of papers has rapidly increased (Figure 1B). China has 3,064 citations and a citation/article ratio of 44.503, ranking first among all selected countries/regions, but its citations per article (9.01) are far lower than that of Canada (29.67). However, Canada's total number of articles (33) and H-index (11) are relatively weak performance. The H-index is a new method for evaluating academic achievements, and a higher H-index indicates that papers are more influential. Combining these paper evaluation indicators, the United States, China, and Canada are the three most influential countries in this research field. From this, we further determined the annual national output of the 10 most productive countries/regions (Figure 1C).

**Table 1:** Top 10 producing countries/regions and institutions related to radiomics in neurological disease research.

**Rank**	**Countries/Regions**	**Articles (*N*)**	**Percentage (*N*)**	**H-index**	**Citations per article**	**Times cited**	**Rank**	**Institutes**	**Articles (*N*)**	**Percentage (*N*)**	**Location**
1	China	340	44.503	31	9.01	3,064	1	Fudan University	45	5.89	China
2	United States	194	25.393	34	19.79	3,839	2	Chinese Academy of Sciences	41	5.366	China
3	Germany	81	10.602	19	16.02	1,298	3	Capital Medical University	38	4.974	China
4	South Korea	51	6.675	18	20.78	1,060	4	General Electric	35	4.581	United States
5	Italy	50	6.545	15	14.14	707	5	Helmholtz Association	34	4.45	Germany
6	France	46	6.021	13	10.5	483	6	Institut National de la
Sante et de la Recherche Medicale INSERM	33	4.319	France
7	United Kingdom	45	5.628	12	18.51	833	7	Institute of Automation CAS	27	3.534	China
8	Netherlands	34	4.45	11	25.18	856	8	UDICE French Research Universities	26	3.403	France
9	Canada	33	4.319	11	29.67	979	9	Harvard University	24	3.141	United States
10	Spain	22	2.88	8	10.55	232	10	University of Chinese Academy of Sciences CAS	24	3.141	China

**Figure 2:**
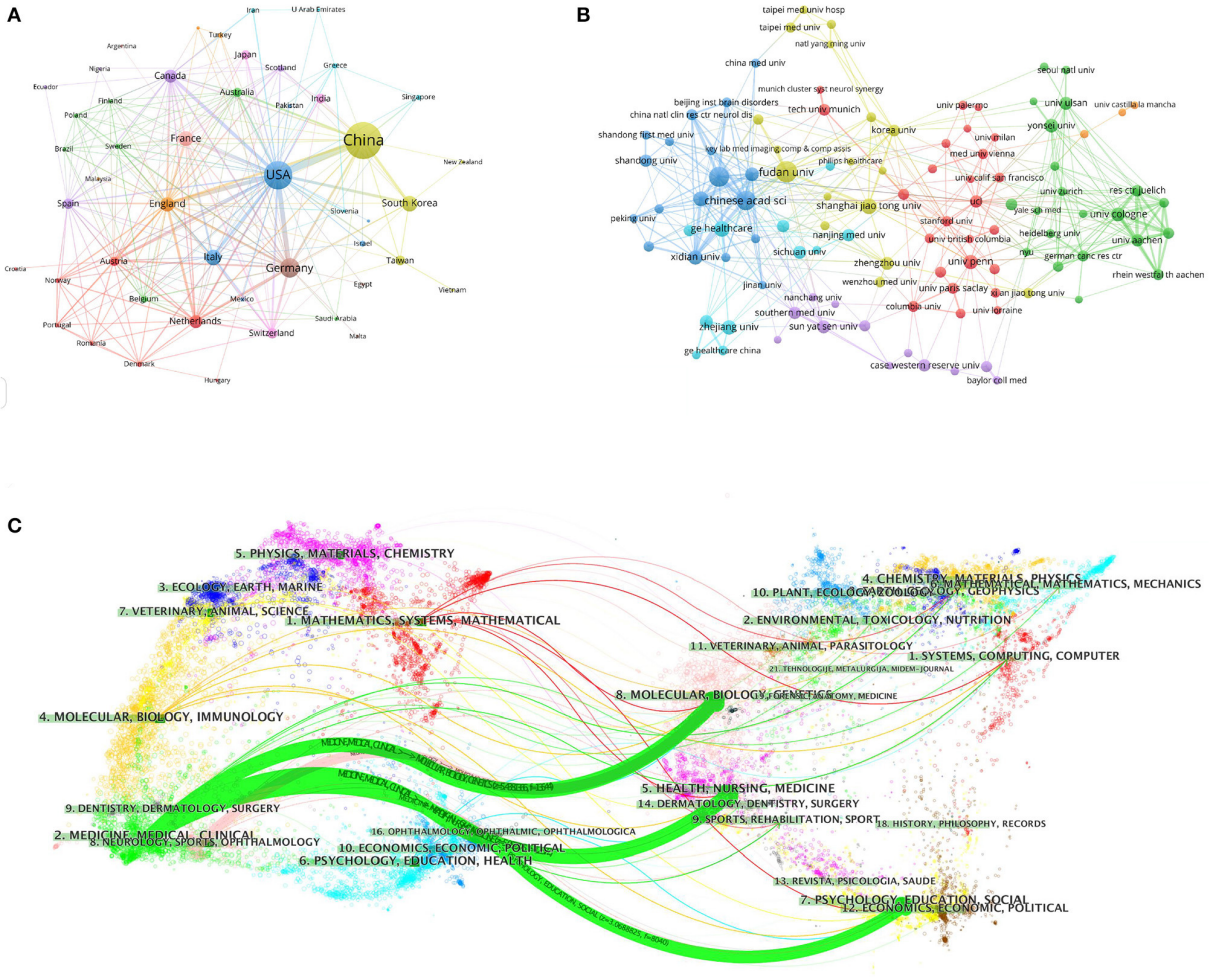
Visualization map of the VOSviewer network of countries/regions and institutions involved in radiomics in neurological diseases. **(A)** Collaboration analysis by country/region. **(B)** Collaboration analysis by agency. **(C)** Double graph superposition of citations of articles on the application of radiomics in neurological diseases (the left side is the cited journal, the right side is the cited journal, and the curve path represents the citation relationship).

To investigate international cooperation, we constructed a network visualization map of publications in radiomics studies of neurological diseases using VOSviewer. Figure 2A displays the collaboration between countries/regions that published more than 10 papers (38 out of 76 papers). Countries/regions with high co-occurrence rates are grouped into the same color. Countries/regions with similar colors are identified as having closer collaboration and forming clusters. The width of the lines represents the scale of cooperation. The United States (220) has the highest total contact strength, indicating its participation in most of the world's collaborations. The countries/regions that cooperate most with the United States are China, Germany, the United Kingdom, Canada, France, and the United States. The yellow cluster is led by China and cooperates most with the United States, Germany, the United Kingdom, and South Korea. As is shown in Figure 2B, there is less cooperation between the most influential countries, and future cooperation should be strengthened to further promote the development of this field. Table 1 shows the 10 most effective institutions in related research. The main institutions are Fudan University (45, 5.89%), the Chinese Academy of Sciences (41, 5.366%), Capital Medical University (38, 4.974%), General Electric (35, 4.581%), and Helmholtz Association (34, 4.45%).

### 3.3. Distribution by journal

A total of 764 publications on brain disease radiomics research have been published in 257 academic journals. Table 2 lists the 10 most productive and frequently cited journals. FRONTIERS IN ONCOLOGY (62 articles, 7.984%) with an IF of 5.78 has the most publishments, followed by EUROPEAN RADIOLOGY (53 articles, 6.937%), CANCERS (34 articles, 6.97%), SCIENTIFIC REPORTS (33 articles, 4.319%), and FRONTIERS IN NEUROSCIENCE (28, 3.665%). The Journal of MAGNETIC RESONANCE IMAGING has the highest IF (13.029) among the top 10 most influential journals in 2019, while EUROPEAN RADIOLOGY has the highest H-index (19). Among the 10 most productive journals, 4 are classified in Q1, 5 in Q2, and one in Q3. The most frequently co-cited journal is RADIOLOGY (552 cited, Q1), with the highest IF (29.146) in 2021. The next most frequently cited journals are EUROPEAN RADIOLOGY (411 cited, Q2), SCIENTIFIC REPORTS (396 cited, Q1), the AMERICAN JOURNAL OF NEURORADIOLOGY (390 cited, Q3), and NEURO ONCOLOGY (379 cited, Q1). The dual map shows three main reference paths. The left side shows the research frontier, with articles concentrated in journals in the medical, neurological, and clinical fields, while the right side shows the cited region, with articles primarily published in journals in the molecular, biological, genetic, psychological, health, nursing, and medical fields (Figure 2C).

**Table 2:** Top 10 productive and co-cited journals for radiomics research in neu authors and co-cited authors.

**Rank**	**Productive journal**	**Articles (*N*)**	**Percentage (*N*)**	**IF (2022)**	**H-index**	**Quartile in category**	**Rank**	**Co-cited journal**	**Articles (*N*)**	**IF (2022)**	**H-index**	**Best quartile**
1	FRONTIERS IN ONCOLOGY	61	7.984	5.738	10	Q2	1	RADIOLOGY	552	29.146	4	Q1
2	EUROPEAN RADIOLOGY	53	6.937	7.034	19	Q1	2	EUROPEAN RADIOLOGY	411	7.034	4	Q1
3	CANCERS	34	4.45	6.575	7	Q1	3	SCIENTIFIC REPORTS	396	4.996	15	Q2
4	SCIENTIFIC REPORTS	33	4.319	4.996	15	Q2	4	AMERICAN JOURNAL OF NEURORADIOLOGY	390	4.966	5	Q2
5	FRONTIERS IN NEUROSCIENCE	28	3.665	5.152	7	Q2	5	NEURO ONCOLOGY	379	13.029	9	Q1
6	FRONTIERS IN NEUROLOGY	16	2.094	4.086	4	Q2	6	PLOS ONE	337	3.752	4	Q2
7	MEDICAL PHYSICS	14	1.832	4.506	5	Q2	7	JOURNAL OF MAGNETIC RESONANCE IMAGING	318	5.119	7	Q1
8	NEURO ONCOLOGY	14	1.832	13.029	9	Q1	8	NEUROIMAGE	266	7.4	1	Q1
9	JOURNAL OF MAGNETIC RESONANCE IMAGING	13	1.702	5.119	7	Q1	9	CLINICAL CANCER RESEARCH	251	13.801	4	Q1
10	NEURORADIOLOGY	13	1.702	2.995	6	Q3	10	MAGNETIC RESONANCE IMAGING	240	3.13	3	Q3

**Figure 3:**
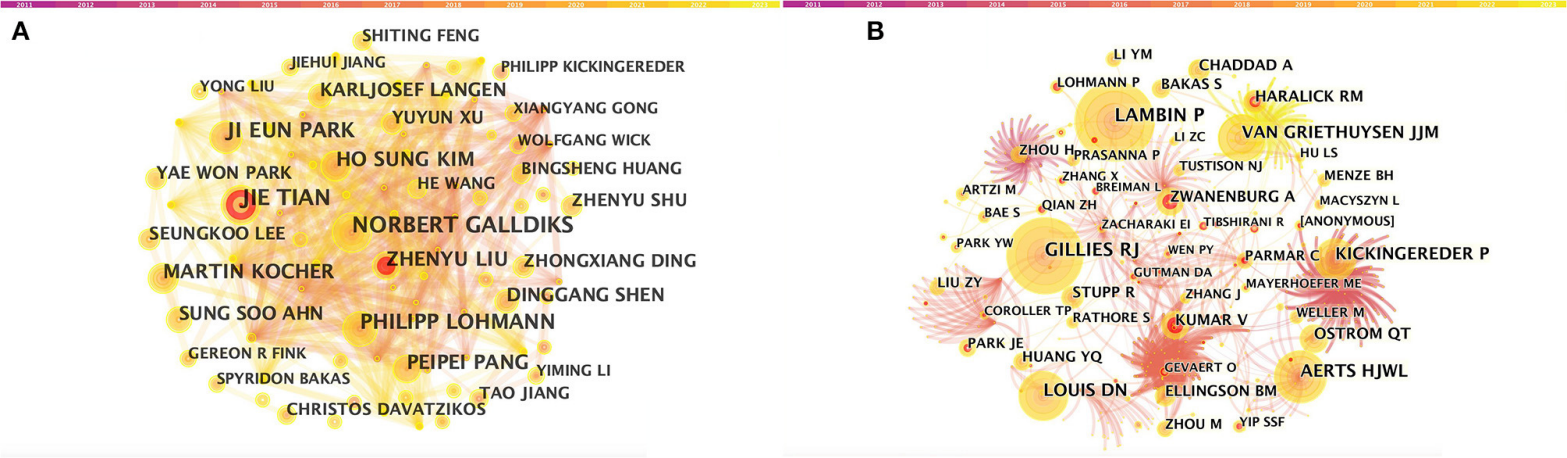
CiteSpace visualization of authors. **(A)** Co-occurrence of authors. **(B)** Co-cited authors involved in radiomics neurological disease research. The circle node represents the author of the paper. Links between nodes represent partnerships.

A total of 5,026 authors participated in the study. Table 3 shows the 11 most productive authors. NORBERT GALLDIKS and JIE TIAN each published 16 articles, ranking first in the number of publications, followed by PHILIPP LOHMANN and JI EUN PARK (13 papers), HO SUNG KIM and MARTIN KOCHER (12 papers), and Zhengyu LIU and ZhengYU LIU (11 papers; Figure 3A and Table 3). It is worth noting that the concentration of authors is relatively low (< 0.03), indicating that the authors' impact on neurological disease radiomics research needs to be increased. In this figure, each node represents an author, the larger the node, the more articles are published. The thick lines indicate close cooperation between authors, as can be clearly seen in Figure 3A, there is communication and cooperation between authors in this field. Co-cited authors refer to those commonly cited in publications, which are critical indicators of author contributions. Figure 3B and Table 3 show the top 11 co-cited authors, with only eight authors having more than 100 times citations, where GILLIES RJ (286) ranked first, followed by LAMBIN P (285), AERTS HJWL (197), and LOUIS DN (185). Upadhaya is an early researcher in the field of neurological system radiomics research.

**Table 3:** Top 11 prolific authors and co-cited authors of radiomics studies in neurological diseases.

**Rank**	**Author**	**Count (*N*)**	**Rank**	**Co-cited author**	**Count (*N*)**
1	NORBERT GALLDIKS	166	1	GILLIES RJ	286
2	JIE TIAN	16	2	LAMBIN P	285
3	PHILIPP LOHMANN	13	3	AERTS HJWL	197
4	JI EUN PARK	13	4	LOUIS DN	185
5	HO SUNG KIM	12	5	Van GRIETHUYSEN JJM	174
6	MARTIN KOCHER	12	6	KICKINGEREDER P	153
7	ZHENYU LIU	11	7	OSTROM QT	106
8	PEIPEI PANG	11	8	KUMAR V	104
9	SUNG SOO AHN	10	9	ZWANENBURG A	98
10	DINGGANG SHEN	9	10	HARALICK RM	92
11	KARLJOSEF LANGEN	9	11	STUPP R	86

Upadhaya is an early researcher of radiomics research in the nervous system. In 2017, he first proposed a prognostic model of glioblastoma multiforme based on multimodal MRI, marking the beginning of radiomics in the field of the nervous system. Meanwhile, Galldiks et al. (40) has published the most articles. In 2020, he summarized the imaging challenges related to immunotherapy, targeted therapy, and brain metastasis combined with radiotherapy. This paper also reviewed advanced imaging techniques that could overcome some of these imaging challenges. It provides valuable information for the identification of changes and recurrences caused by treatment in brain metastasis lesions, and the evaluation of treatment responses. In the same year, Kim et al. (41) proposed a method using diffusion and perfusion-weighted MRI radiomics models to predict isocitrate dehydrogenase (IDH) mutation and tumor aggressiveness in diffuse low-grade gliomas. In this paper, multiparameter MRI radiomics models are also utilized to predict tumor grades.

In 2021, PHILIPP LOHMANN reviewed the basics, current workflows, and methods of radiomics, and focused on the application of feature-based radiomics in neural tumors with clinical examples. Additionally, he studied the usage of FET-PET radiomics in identifying glioma relapse and progression (42, 43). Park et al. (44) is a senior researcher in the neurological disease radiomics field. By using deep learning to automatically segment diffusion and perfusion MRI radiomics, he provided a reproducible and comparable diagnostic model for glioblastoma. He concluded that the first-level feature extraction based on automatically segmented MRI has high reproducibility and comparable diagnostic efficiency with manual segmentation. This field remains a key focus of radiomics in the research of nervous system diseases.

### 3.4. Analysis of co-cited references

**Figure 4:**
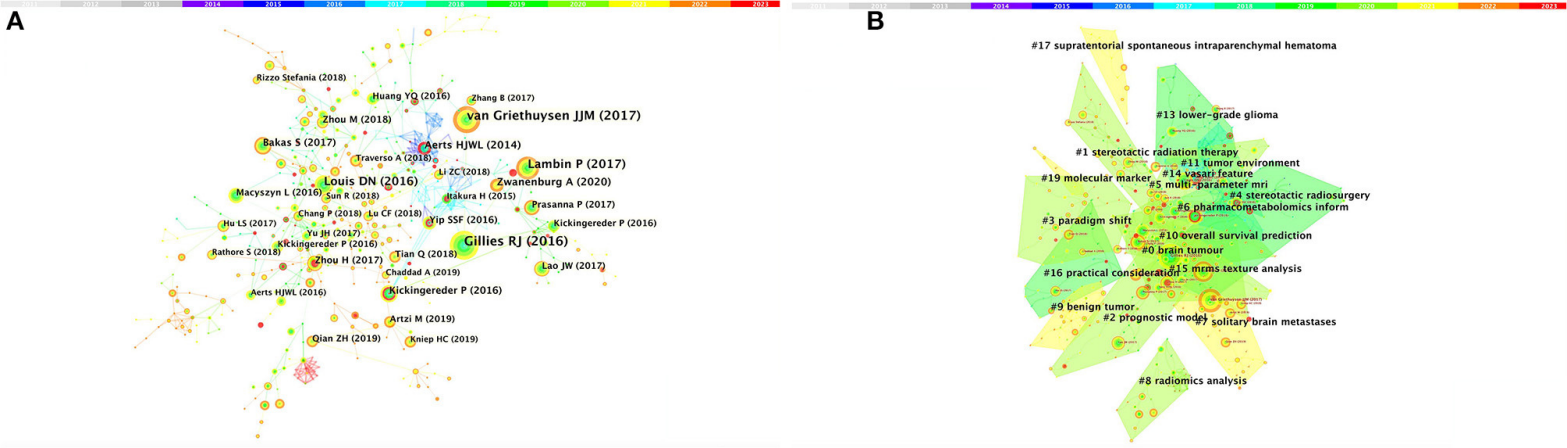
Analysis of references related to radiomics in the study of neurological diseases. **(A)** Network of co-cited references. **(B)** Network diagram of co-reference clusters.

Among the top 10 co-cited articles, four were critical articles, two were on radiomics, and four were on nervous system diseases. Additionally, these studies are considered reliable references for future relevant research. It's worth noting that among the top five co-cited references, there are no articles about radiomics research in the nervous system, which indicates that the field still requires further research (Figure 4A and Table 4). The network diagram of co-reference clusters is shown in Figure 4B, and the data collection is shown in Table 5. In 2014, Aerts and HJWL extracted 440 features from computer tomography scan data of 1,019 lung or head or neck cancer patients, and conducted radiology analysis on the image intensity, shape, and texture of the tumors. The results concluded that a large number of radiomics features have predictive capabilities in an independent dataset of cancer and head and neck cancer patients. He also proposed the concept of radiomics genomics and showed that radiomics genomics features can capture tumor heterogeneity with underlying gene expression patterns (45). This study is considered a significant milestone and marks the transition of radiomics genomics from basic research to clinical application. Kickingereder et al. (46) demonstrated that compared to established clinical radiomics risk models, radiomics-based magnetic resonance imaging signals can improve the accuracy of predicting survival and stratification of newly diagnosed glioblastoma patients.

**Table 4:** 10 commonly cited literatures in imageomics research of nervous system diseases.

**Rank**	**Co-citations (*N*)**	**Centrality**	**First author**	**Year**	**Title**	**Journal**	**DOI**	**Cluster**
1	218	0.01	Gillies, RJ	2016	Radiomics: images are more than pictures, they are data (survey)	Radiology	10.1148/radiol.2015151169	#4
2	176	0	Van Griethuysen, JJM	2017	Computational radiomics system to decode the radiographic phenotype	Cancer Research	10.1158/0008-5472.CAN-17-0339	#4
3	134	0.02	Lambin, P	2017	Radiomics: the bridge between medical imaging and personalized medicine (survey)	Nature Reviews Clinical Oncology	10.1038/nrclinonc.2017.141	#4
4	114	0.01	Louis, DN	2016	The 2016 World Health Organization classification of tumors of the central nervous system: a summary	Acta Neuropathologica	10.1007/s00401-016-1545-1	#4
5	70	0.13	Aerts, HJWL	2014	Decoding tumor phenotype by noninvasive imaging using a quantitative radiomics approach	Nature Communications	10.1038/ncomms5006	#4
6	66	0	Aerts, HJWL	2020	The image biomarker standardization initiative: standardized quantitative Radiomics for high-throughput image- based phenotyping	Radiology	10.1148/radiol.2020191145	#2
7	56	0.1	Kickingereder, P	2016	Radiomic profiling of glioblastoma: identifying an imaging predictor of patient survival with improved Performance over established clinical and radiologic risk models	Radiology	10.1148/radiol.2016160845	#3
8	54	0.01	Bakas, S	2017	Data descriptor: advancing the cancer genome atlas glioma MRI collections with expert segmentation labels and radiomic features	Scientific data	10.1038/sdata.2017.117	#4
9	45	0.04	Yip, SSF	2016	Applications and limitations of radiomics (survey)	Physics in medicine and biology	10.1088/0031-9155/61/13/R150	#4
10	44	0.27	Zhou, H	2017	MRI features predict survival and molecular markers in diffuse lower-grade gliomas	Neuro-oncology	10.1093/neuonc/now256	#3

**Table 5:** Top 10 co-cited reference clusters for radiomics studies in neurological diseases.

**Cluster ID**	**Size**	**Silhouette**	**Mean (Year)**	**Top terms**
0	40	0.896	2016	Glioma imaging
1	40	0.915	2017	Radiotherapy
2	34	0.947	2016	Prognostic biomarker
3	32	0.982	2018	Tumor aggressiveness
4	32	0.992	2014	Stereotactic radiosurgery
5	31	0.921	2016	Multiple pathologic biomarkers
6	29	0.958	2014	Risk stratification
7	29	0.975	2019	Glioblastoma
8	29	0.994	2018	Radiomics classification
9	29	0.959	2018	Machine learning applications

In 2017, Bakas et al. (47) collected the segmentation labels and radiological features of preoperative multimodal magnetic resonance imaging (MRI) of gliomas from multiple institutions in the Cancer Genome Atlas (TCGA), and collected stratified gliomas by preoperative scans. The significant value of the study lies in the fact that the data is publicly available. The generated labels and imaging features can be used for repeatable and comparable quantitative studies, providing guidance for addressing reproducibility and feature interpretation problems. Van Griethuysen et al. (48) proposed a theory that the lack of standardized definitions and image processing severely affects the reproducibility and comparability of research results. They developed a flexible open-source platform PyRadiomics and discussed the workflow and architecture of PyRadiomics. This study demonstrates its application in the feature analysis of lung lesions which is expected to address the standardization problem of algorithms and image processing. In 2020, Zwanenburg et al. (18) proposed the Image Bio-Marker Standardization Initiative and standardized 174 radiomics features, providing a foundation for the validation and calibration of different radiomics software. It also provided an effective solution to the current common data island problem. Despite the involvement of a large number of literature in the feature standardization study, it still lacks a better solution.

### 3.5. Analysis of keyword co-occurrenceclusters

**Figure 5:**
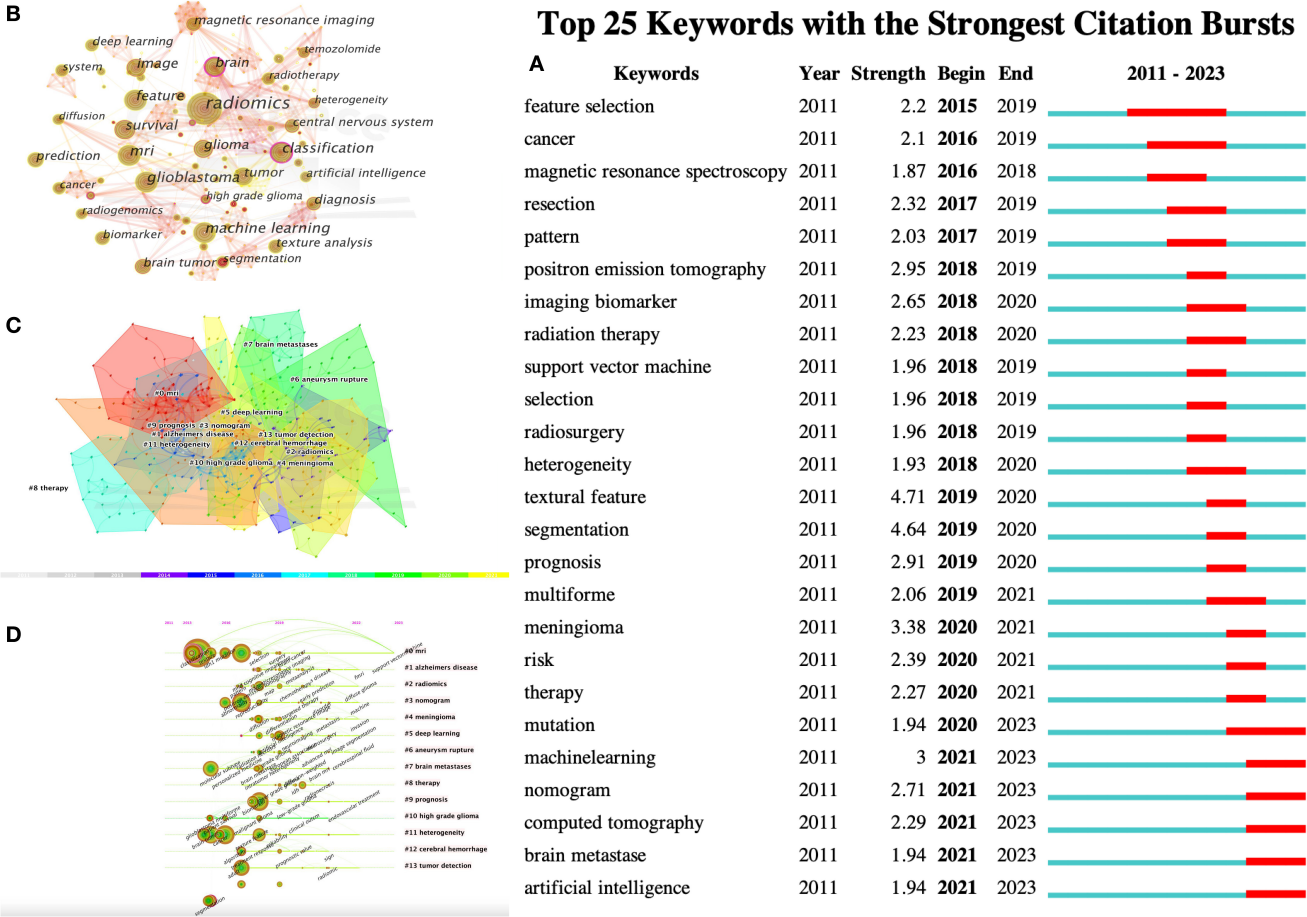
CiteSpace visualization of keywords in radiomics studies of neurological diseases. **(A)** Key keywords network in radiomics neurological disease research and related research publications. **(B)** Time trend plot of emergent keywords. **(C)** Keyword cluster analysis. **(D)** Timeline view of Keywordclusters.

Keywords were condensed and extracted from the content of an article to reflect the topic and content of a representative article. High-frequency keywords are often used to reflect the hot topics in the research field. Thus, a keyword co-occurrence network is an analysis method based on text content. The authors collect 383 keywords (Figure 5A) and find the top 25 most frequently cited keywords through keyword burst analysis (Figure 5B). The blue line represents the time interval, and the red line represents the burst period of the keywords. As can be seen from the figure, the burst keywords are mainly concentrated on the research of radiomics and brain-related diseases, including but not limited to disease risk analysis, treatment, and tumor heterogeneity. Feature selection, patterns, magnetic resonance spectroscopy analysis, and image segmentation are the pioneering explorations in the early stage. Since then, positron emission tomography (PET), imaging biomarkers, radiotherapy, support vector machines, feature selection, tumor heterogeneity, image texture features, and disease prognostic evaluation became hot topics. In recent years, computed tomography (CT), nomograms, brain metastasis, machine learning, and gene mutations have become new key keywords for outbreaks.

The results in Figure 5C calculated by the log-likelihood ratio (LLR) show the keyword radiation. The clustering analysis shows outstanding homogeneity through 17 cluster centers with a modularity score of 0.781 and an average silhouette value of 0.8346. They encompass a wide range of radiomics topics in the field of neurological diseases, including artificial intelligence and imaging techniques [# 0 mri (32), # 2 radiomics (32), # 3 nomogram (33), and # 5 deep learning (33)] and indications [# 1 Alzheimer's disease (34), # 4 Meningioma (35), # 6 ruptured aneurysm (36), # 7 brain metastases (37), # 8 treatment (38), # 9 prognosis (38), # 10 high-grade glioma (38), # 11 tumor heterogeneity (38), # 12 intracerebral hemorrhage (39), and # 13 tumor detection (39); Figures 5C, D]. In recent years, the advancement of AI technology has significantly improved the status of radiomics research, making it a hot topic in the field of the combination of medicine and engineering. This has brought new opportunities for clinical diagnosis and treatment. Moreover, radiomics has also been proven to have good performance in the diagnosis, prediction, and prognosis of nervous system diseases. Recent researches are more focused on the relationship between radiomics markers and the internal microenvironment of nervous system diseases, particularly the correlation between tumor heterogeneity, immunity, metabolism, and imaging markers. The reproducibility and reliability of the models have always been a focus of research and are expected to be solved in the future with further development of wireless technology.

## 4. Discussion

In recent years, increasing numbers of systematic and narrative reviews have focused on the application of radiomics in the study of nervous system diseases (49–52). The use of radiomics techniques is highlighted in image segmentation, prognosis, and non-invasive biomarker applications. An advantage of bibliometrics analysis compared to traditional surveys is that it provides the reader with an intuitive visualization of the research situation on a particular topic. Here, we conducted a novel bibliometric analysis to explore the publications of the past decade and provide a comprehensive view of research trends.

As the concept of radiomics was first proposed in 2012, we searched the data from 2012. The sudden rapid growth is related to the magnetic resonance imaging features proposed by Zhou et al. (53) that it can be used to predict the survival time and molecular distribution of low-grade gliomas (LGGs). Texture analysis of MRI data can accurately predict IDH1 mutation, 1p/19q co-deletion, histological grading, and tumor progression. This highly cited study is considered a leap in radiomics from the macroscopic features of gliomas to the microscopic internal environment, greatly driving the research in this field. A machine learning model based on morphological features derived from Pyradiomics was used to predict aneurysm stability (54), and this marked the beginning of widespread research on radiomics in the field of nervous system diseases. Subsequently, Tupe-Waghmare et al. (55) proposed that compared to traditional risk factors, radiomics models based on deep learning performed better in predicting the survival rate of glioblastoma multiforme. To further this work, they proposed a radiomics nomogram, which proved its good predictive performance and showed that multi-variable models have statistical robustness in survival analysis (56–59). In this section, the narrative logic will unfold from two aspects, traditional explorations and deep learning processing.

First we analyze the traditional explorations and traditional imaging techiniques. Since 2018, a large number of radiomics studies in the field of nervous system diseases have emerged, including glioma survival analysis and stratified prediction (60), the differential diagnosis of gliomas (61), and gene mutations (62). Meanwhile, the research on Alzheimer's disease is also growing (63). At the same time, the use of multi-parametric MRI to predict the IDH mutation status in glioblastoma (GBM) by employing multi-region radiomics features was also been discussed (64). The results showed that the multi-region model constructed by the whole-region features performed better than the single-region model, and the best performance was achieved when combined with the age-whole-region. Meanwhile, the research team made another attempt on predicting the O-6-methylguanine-DNA methyltransferase (MGMT) promoter methylation status of GBM based on a multi-region and multi-parametric MRI radiomics model, and the results showed that combining clinical factors with radiomics features does not improve predictive performance (65).

Another exploratory work provided by Bobholz et al. (66) investigated the local relationship between MR-derived radiomics features and histology-derived “tissue” features using a dataset of 16 brain cancer patients. Radiomics features were collected from T1, post-contrast T1, FLAIR, and diffusion-weighted imaging (DWI) acquired before death. Similar tissue features were collected from autopsy samples and registered via magnetic resonance imaging. The results showed that a subset of radiomics features can consistently capture texture information of histological tissue. Su et al. (56) combined eight imaging features and three clinical variables (age, sex, and tumor location) to construct an imaging omic-clinical nomogram. The nomogram presented good discrimination in predicting the isocitrate dehydrogenase 1 (IDH1) mutation status of primary GBMs. On the other hand, Sakai et al. (67) found that the XGBoost model trained on DWI data can achieve an accuracy of 90% in predicting IDH1 mutation status, but the model trained based on combined FLAIR-DWI radiomics features could not improve the accuracy.

Following these ideas, the research became more detailed, including automatic image segmentation (68), multiple sclerosis (31), application of PET radiomics (69), and more advanced non-invasive identification of gene mutations (70). Guo et al. (2) investigated the role of Dsc-PWI dynamic radiomics features in the diagnosis and prognosis prediction of ischemic stroke. Cao et al. (71) used the radiomics features of MRI and 18F-FDG-PET and the joint application of multiple models to identify gliomas. Alongi et al. (72) used AI-based 18F-FDG-PET to improve the accuracy of AD diagnosis. Yao et al. (73) evaluated the ability of pH and oxygen-sensitive MRI techniques to differentiate glioma genotypes and concluded that pH and oxygen-sensitive MRI is a viable and potentially valuable imaging technology to distinguish glioma subtypes and provide additional prognostic value in clinical practice. Dounavi et al. (74) suggested that FLAIR texture analysis can capture subtle alterations in white matter microstructural.

To analyze the deep learning processing part, one extraordinary method provided by Calabrese et al. (75) combined radiomics features and convolutional neural network (CNN) features, and the collaborative model performed well in predicting IDH1 and TERT promoter hotspot mutations, ATRX and CDKN2A/B pathogenic mutations, and chromosome 7 and 10 combined aneuploidy, but not well in predicting other biomarkers. The hybrid CNN-Transformer encoder based on multimodal MRI proposed by Cheng et al. (76) achieved good performance in both glioma segmentation and IDH gene typing prediction, outperforming single-task learning and other state-of-the-art methods.

Previous studies have found that the use of radiomics features as biomarkers of treatment response and outcome may be correlated with clinical phenotypes, histological features, and genomic features, but robust and reproducible features are needed to address this issue. The low replicability potential of the current study is still the reason why radiomics-based strategies have not yet been translated into routine practice. Tixier et al. (77) found that the robustness of radiomics features varied by category and features calculated based on Gray-level Size-zone Matrix (GLSZM), edge maps, and shape were less robust compared to histograms and co-occurrence matrics. Ma et al. (78) showed that first-order and GLCM features extracted from LoG and wavelet-filtered images were the most crucial factors for glioma recognition. Additionally, some eigenvalues were considered strongly correlated between low-grade glioma (LGG) and high-grade glioma (HGG).

Since plenty of scholars have been involved in studying the robust performance of deep learning feature models, the results are satisfactory compared to traditional machine learning models. However, the results of scientific research are still questionable because the performance of deep learning methods depends on high-level data processing capability and high-quality data requirements. On the other hand, the internal algorithm feature vectors in unsupervised deep learning may not always be transparent (black box) (79). It is worth illustrating that the research of feature reproducibility involves multiple aspects, including support from multi-center data and further research on image segmentation. Moreover, the optimal feature selection methods and the non-uniformity and standardization of features are also urgent problems to be solved.

### 4.1. Limitations

To the best of our knowledge, this bibliometric analysis is the first exploration of the development and trends of radiomics research in the field of neurological diseases. However, this study has some limitations. Firstly, the data used in this study was extracted only from the WoSCC database, as we believe this database is a reliable software for searching publications and citations, although it may contain fewer documents and journals than other databases such as Google Scholar or Scopus. Secondly, non-English articles were excluded from the database and analysis, which may have led to a source bias. Additionally, we selectively analyzed the characteristics of information, so some key points and details may have been missed.

## 5. Conclusion

Bibliometrics analysis indicates a good prospect and a significant increase in related publications for radiomics in the field of neurological diseases. The main contributors to this research area have been identified, and the related studies are clustered and mainly focused on the application of radiomics in glioma, brain metastasis, and white matter diseases. Differences between regions and countries still exist, and cooperation between countries needs to be strengthened. In the process of applying deep learning for handling radiomics data, there exists such a problem that the data is in a poorly interpreted form of image features, but this problem will be gradually solved with the advancement of technology. By using these methods, it helps better understand the prospects of radiomics in neurological diseases, and facilitates clinical diagnosis and treatment decisions, simplifies the diagnostic and therapeutic process, and ultimately benefits patients.

## Data availability statement

The data was downloaded from the Science Citation Index Extended database of the Web of Science Core (WoSCC) on October 26, 2022. The following search terms were used: (“radiomics”) and (“brain” or “cerebrum” or “encephalon” or “pericranium” or “cerebral^*^” or “central nervous system”) in the Topic field, including title, abstract, author keywords, and KeyWords Plus. Original articles and reviews written and published in English between 2011 and 2022 were included. The result of the survey was 764 records, which were obtained from this study.

## Author contributions

JC: data analysis and writing. XM: methodology. XY: validation. XQ: formal analysis. All authors contributed to the article and approved the submitted version.
